# Association of thrombocytopenia and D-dimer elevation with in-hospital mortality in acute aortic dissection

**DOI:** 10.1080/07853890.2025.2478477

**Published:** 2025-03-21

**Authors:** Xingwei He, Abudunaibi Balati, Wenhua Wang, Hongjie Wang, Baoquan Zhang, Chunwen Li, Dan Yu, Suping Guo, Hesong Zeng

**Affiliations:** ^a^ Department of Cardiology, Tongji Hospital, Tongji Medical College, Huazhong University of Science and Technology, Wuhan, China; ^b^ Hubei Provincial Engineering Research Center of Vascular Interventional Therapy, Wuhan, China; ^c^ Department of Cardiac Intensive Care Unit, Central China Fuwai Hospital of Zhengzhou University (Fuwai Central China Cardiovascular Hospital), Zhengzhou, China; ^d^ Department of Cardiology, Tongji Xianning Hospital, Xianning, China; ^e^ Department of Critical Care Medicine, The Third Affiliated Hospital of Xinxiang Medical University, Xinxiang, China; ^f^ Department of Emergency Medicine, The Second Affiliated Hospital of Chongqing Medical University, Chongqing, China; ^g^ Department of Cardiac Intensive Care Unit, People’s Hospital of Zhengzhou University (Henan Provincial People’s Hospital), Zhengzhou, China

**Keywords:** Acute aortic dissection, thrombocytopenia, D-dimer, platelet, in-hospital mortality

## Abstract

**Background:**

Data on the association between the degree of platelet and coagulative-fibrinolytic variables abnormalities and the risk of in-hospital mortality in acute aortic dissection (AAD) are limited.

**Materials and methods:**

This multicentre retrospective cohort study included patients diagnosed with AAD by aortic computed tomographic angiography between 2010 and 2021 in five tertiary hospitals in China. The primary outcome was defined as all-cause mortality during hospitalization. Associations between platelet counts, coagulation-fibrinolytic parameters and all-cause in-hospital mortality were assessed using Cox proportional hazards regression models.

**Results:**

Among the 2567 participants, the median age was 54 years (interquartile range, IQR: 47–63); 531 (20.7%) were female, and the in-hospital mortality rate was 589 (23.0%). The Cox proportional hazards regression model indicated that lower platelet count, prothrombin activity (PTA), and fibrinogen levels and longer prothrombin time (PT) and thrombin time (TT) were linearly positively associated with an increased risk of in-hospital mortality (*p* < 0.05). A non-linear and positive association was confirmed between D-dimer levels and in-hospital mortality risk (*p* < 0.05). Additionally, a significant interaction between platelet counts and D-dimer levels was observed (*p* = 0.029). According to the subgroup analysis, compared to those in the reference group, those with thrombocytopenia (<140 × 10^9^/L) and high D-dimer levels (>14.6 µg/mL) had a 3.59-fold increased risk of in-hospital mortality (HR, 3.59; 95% CI, 2.00–6.42).

**Conclusions:**

Our analysis revealed associations between changes in platelet count, PT, PTA, TT, fibrinogen and D-dimer levels and outcomes in patients with AAD. Furthermore, the combined effect of thrombocytopenia and high D-dimer levels significantly increased the risk of in-hospital mortality.

## KEY MESSAGES

Platelet count and coagulation-fibrinolysis system variables, including PT, PTA, TT, fibrinogen and D-dimer levels, are significantly associated with in-hospital mortality risk in acute aortic dissection (AAD) patients.

A significant interaction between platelet count and D-dimer level (*p* = 0.029) highlights that the combined effect of thrombocytopenia and elevated D-dimer levels significantly increases the risk of in-hospital mortality in AAD patients.

Prompt and repeated assessment of platelet counts and coagulation-fibrinolysis system variables upon admission can serve as pivotal biomarkers for risk stratification in AAD patients, facilitating timely and targeted interventions.

## Introduction

Acute aortic dissection (AAD) is a life-threatening cardiovascular disease [1–3]. Despite advances in prompt diagnosis and management, the early mortality rate of AAD remains high. As per the International Registry of Acute Aortic Dissection (IRAD), the reported in-hospital mortality rates are 22% for type A and 12%–14% for type B aortic dissection [4]. Therefore, the ability to, promptly and efficiently assess the risk of mortality from this disorder in its early stages would undoubtedly improve the prognosis of patients diagnosed with AAD in the future.

Platelets and the coagulation-fibrinolysis system play important roles in the occurrence and development of cardiovascular diseases and are often studied to evaluate the prognosis of cardiovascular diseases [5–8]. Changes in the coagulation and fibrinolytic system are always accompanied by changes in the occurrence, development and outcome of AAD. After the intimal tear in the aorta and exposure of collagen, platelet activation, along with the release of tissue factor and thrombosis in the false lumen, triggers the activation of both extrinsic and intrinsic pathways in the coagulation cascade reaction. Subsequently, fibrinolytic systems are also activated, and cross-linked fibrin is degraded [9–12].

There is considerable evidence that shows the relationship between changes in coagulative and fibrinolytic variables, such as plasma fibrinogen, International Normalized Ratio (INR), and D-dimer and prognosis in AAD patients [10,13–16]. However, whether coagulation and fibrinolytic system changes could provide valuable clinical predictive information for mortality in AAD requires further systematic investigation. Most studies concentrate on analyzing only one or two variables of the coagulation and fibrinolysis system, rather than offering a comprehensive assessment. The statistical power in some studies was limited [14,17], and certain results have been met with controversy [14,15]. In addition, as platelet activation plays an essential role in the AAD process, it is still unknown whether there is an inherent interaction between platelets and abnormally activated coagulation and fibrinolytic system variables, thereby providing better predictive value for the prognosis of AAD patients.

Hence, this study aimed to conduct a multicentre retrospective cohort study to clarify the associations among platelet count, coagulation-fibrinolysis system variables and the risk of mortality in AAD.

## Materials and methods

### Study population

We conducted a multicentre retrospective cohort study based on data extracted from the electronic medical records (EMRs) of patients admitted to five tertiary hospitals in China. These hospitals include Tongji Hospital, Tongji Medical College of Huazhong University of Science and Technology; People’s Hospital of Zhengzhou University (Henan Provincial People’s Hospital); Central China Fuwai Hospital of Zhengzhou University (Fuwai Central China Cardiovascular Hospital); The Third Affiliated Hospital of Xinxiang Medical University; and The Second Affiliated Hospital of Chongqing Medical University. Adhering to the Strengthening the Reporting of Observational Studies in Epidemiology (STROBE) guidelines for cohort studies, we enrolled all eligible patients who underwent aortic computed tomographic angiography (CTA) between August 2010 and December 2021 based on the specific EMR situation at various hospitals. Individuals diagnosed with intramural haematoma and penetrating atherosclerotic ulcer, those with aortic aneurysms, postoperative reviews related to aortic dissection, and those without diagnosed aortic diseases were excluded from the study. In addition, patients who missed EMRs or had inadequate EMRs were excluded. After applying the first two-round study exclusion criteria, the enrolled AAD patients were screened for further extraction of study variables. Finally, patients under 18 years of age, during pregnancy, minority nationality, with incomplete clinical data, and non-acute phase were further excluded after the variables were collected (Supplementary Figure S1 [18]).

This research was approved by the Research Ethics Commissions of Tongji Hospital Tongji Medical College of Huazhong University of Science and Technology (TJ-IRB20211102), People’s Hospital of Zhengzhou University (Henan Provincial People’s Hospital) (2021-190), Central China Fuwai Hospital of Zhengzhou University (Fuwai Central China Cardiovascular Hospital) (2021-38), The Third Affiliated Hospital of Xinxiang Medical University (K2021-039-01), and The Second Affiliated Hospital of Chongqing Medical University (2022-15), respectively, with informed consent waived by the Ethics Commissions aforementioned above. The study was conducted in accordance with the principles of the Declaration of Helsinki.

### Data collection

The variables of demographics, initial vital signs, medical history, routine blood tests, coagulation function, biochemical tests, therapy method, and the status of all-cause in-hospital death were identified based on the records of staff in different hospitals. All analysis results of routine blood tests, coagulation function tests, and biochemical tests were obtained from the laboratory department of each hospital, and all blood test samples were collected within 2 h of admission.

### Definitions

Aortic dissection is characterized by disruption of the medial layer due to various causes, leading to separation of the aortic wall layers and subsequent formation of both a true lumen and a false lumen. When the initial symptoms occur within 14 days, it is classified as AAD [19]. Hypertension was established by a clinical record of systolic blood pressure ≥ 140 mmHg and/or diastolic blood pressure ≥ 90 mmHg or the use of antihypertensive agents [20]. Diabetes mellitus was defined as being on oral medication or insulin for diabetes or a fasting glucose level ≥126 mg/dL [21]. Smoking status was defined when the subjects were current smokers according to self-report.

### Endpoint

The study endpoint was defined as all-cause mortality during hospitalization.

### Statistical analysis

Patient demographic and clinical data are expressed as continuous variables and are represented as medians and intra-quartile ranges (IQRs), and categorical variables are presented as percentages. Spearman’s correlation was employed to assess the relationships among 54 biological variables, and the correlation intensity was determined under the guidance of absolute values. Hierarchical clustering analysis (HCA) was conducted to unveil clustering effects and identify biological variables exhibiting similar patterns. The adjusted hazard ratios (HRs) and 95% confidence intervals (CIs) for in-hospital mortality of AAD were calculated using Cox proportional hazards regression after checking the proportional hazards assumption. Model 1 included only biological variables (unadjusted), Model 2 included biological variables, age, and sex, while Model 3 expanded on Model 2 by adding smoking history, hypertension history, diabetes history, anatomical classification, onset time, and hospital centres. For the restricted cubic spline, knots were positioned at the 10th, 50th and 90th percentiles of the predictor variables, guided by Harrell’s recommended method based on their distribution [22]. Moreover, interaction tests were conducted between the platelet level and the coagulative variables, along with the fibrinolytic variables using Cox regression models. The models were further stratified based on platelet and D-dimer levels in subgroup analyses to estimate the impact of different subgroups on the in-hospital mortality of AAD. Additionally, several sensitivity analyses were performed to test the robustness of our results. Stratified analyses were conducted based on age, sex, smoking history, hypertension history and onset time. Interaction *p*-values were calculated to assess the consistency of results. Additionally, outcomes were analyzed within prespecified subgroups, considering factors such as age, sex, smoking and hypertension history, diabetes history, anatomical classification, onset time, and hospital centres’ influence on AAD mortality. Cox proportional hazard regression models were then re-fitted in the matched population to ensure the stability of results. SAS version 9.4 (SAS Institute, USA) and R statistical software (the R Foundation, http://www.r-project.org, version 4.0.2) were utilized for analyses and plotting with a two-sided significance threshold of *p* < 0.05.

## Results

### Study population

We identified a total of 39,161 patients from five tertiary hospital centres who underwent aortic CTA examinations spanning from August 2010 to December 2021. In the first round of study participant exclusion, 5337 patients were collected as those diagnosed with first-onset AAD from all five hospitals. Moreover, 2925 AAD inpatients with available EMRs were summarized after the second-round exclusion. In total, 2567 patients were confirmed eligible for statistical analysis after the final round of exclusions. A flowchart of the study, including the details of participants’ screening, is shown in Supplementary Figure S1 [18].

### Baseline characteristics

The baseline characteristics of the study participants across platelet-level categories are listed in Table 1. Among the 2567 participants included, the median (IQR) age was 54 (47–63) years, and 531 (20.7%) were women. Compared with participants with a higher platelet count, those with a lower platelet count were more likely to be older and exhibit a higher prevalence of DeBakey I anatomical classification for AAD. Furthermore, these patients more frequently presented with abnormal laboratory findings, including elevated Activated Partial Thromboplastin Time (APTT), Thrombin Time (TT), Prothrombin Time (PT) and D-dimer levels, with median values (IQRs) of 38.7 (35.1–42.9), 17.0 (15.8–18.8), 14.6 (13.8–15.7) and 13.6 (4.2–21.0), respectively. Additionally, Prothrombin Activity (PTA) and fibrinogen levels are lower, with median values (IQRs) of 79.0 (69.0–89.0) and 2.4 (1.8–3.3), respectively. Moreover, individuals with lower platelet levels exhibit an increased in-hospital mortality in AAD. The baseline characteristics of patients stratified by D-dimer levels were similar (see Supplementary Table S1).

**Table 1.** Baseline characteristics of patients with AAD stratified by platelet levels.

Characteristics	≤140 × 10^9^/L	140∼186 × 10^9^/L	≥186 × 10^9^/L
Age, median (IQR), years	58.0 (50.0, 66.0)	54.0 (47.0, 61.0)	51.0 (43.0, 60.0)
Sex, Male, n (%)	665 (78.5%)	680 (81.1%)	655 (78.0%)
Anatomical classification			
DeBakey I, n (%)	490 (57.9%)	403 (48.1%)	337 (40.1%)
DeBakey II, n (%)	43 (5.1%)	47 (5.6%)	56 (6.7%)
DeBakey IIIa, n (%)	22 (2.6%)	19 (2.3%)	32 (3.8%)
DeBakey IIIb, n (%)	265 (31.3%)	343 (40.9%)	372 (44.3%)
Isolated abdominal AAD, n (%)	27 (3.2%)	26 (3.1%)	43 (5.1%)
History			
Smoking, n (%)	325 (38.4%)	338 (40.3%)	357 (42.5%)
Hypertension, n (%)	560 (66.1%)	562 (67.1%)	601 (71.5%)
Diabetes, n (%)	23 (2.7%)	24 (2.9%)	34 (4.0%)
Larger aorta diameter (≥5.5 cm), n (%)	18 (2.1%)	16 (1.9%)	26 (3.1%)
Onset time			
<24 h, n (%)	517 (61.0%)	536 (64.0%)	452 (53.8%)
1–7 days, n (%)	309 (36.5%)	286 (34.1%)	310 (36.9%)
8–14 days, n (%)	21 (2.5%)	16 (1.9%)	78 (9.3%)
Hospital centres, n (%)			
Tongji Hospital	673 (79.5%)	670 (80.0%)	600 (71.4%)
Henan Provincial People’s Hospital	75 (8.9%)	89 (10.6%)	126 (15.0%)
Fuwai Central China Cardiovascular Hospital	35 (4.1%)	31 (3.7%)	50 (6.0%)
Third Affiliated Hospital of Xinxiang Medical University	16 (1.9%)	18 (2.1%)	29 (3.5%)
Second Affiliated Hospital of Chongqing Medical University	48 (5.7%)	30 (3.6%)	35 (4.2%)
Hospital mortality, n (%)	235 (27.7%)	190 (22.7%)	144 (17.1%)
Hospitalization time, median (IQR), days	10.0 (3.0, 20.0)	12.0 (4.0, 21.0)	12.0 (5.5, 21.0)
Plateletcrit, median (IQR), %	0.1 (0.1, 0.1)	0.2 (0.2, 0.2)	0.2 (0.2, 0.3)
APTT, median (IQR), s	38.7 (35.1, 42.9)	37.0 (33.6, 40.4)	36.3 (32.9, 40.0)
TT, median (IQR), s	17.0 (15.8, 18.8)	16.7 (15.7, 18.0)	16.4 (15.5, 17.4)
PT, median (IQR), s	14.6 (13.8, 15.7)	14.1 (13.3, 14.8)	13.7 (12.8, 14.4)
PTA, median (IQR), %	79.0 (69.0, 89.0)	86.0 (76.0, 95.0)	89.2 (81.0, 101.0)
INR, median (IQR)	1.2 (1.1, 1.3)	1.1 (1.0, 1.2)	1.1 (1.0, 1.1)
Fibrinogen, median (IQR), g/L	2.4 (1.8, 3.3)	2.6 (2.1, 3.4)	3.2 (2.5, 4.9)
D-dimer, median (IQR), µg/mL	13.6 (4.2, 21.0)	7.8 (2.9, 21.0)	3.5 (1.5, 9.7)

Continuous variables are represented as median (IQR) and categorical variables as number (%).

IQR: interquartile range; AAD: acute aortic dissection; APTT: activated partial thromboplastin time; TT: thrombin time; PT: prothrombin time; PTA: prothrombin activity; INR: International Normalized Ratio.

### Correlation analysis and hierarchical cluster analysis

**Figure 1.**
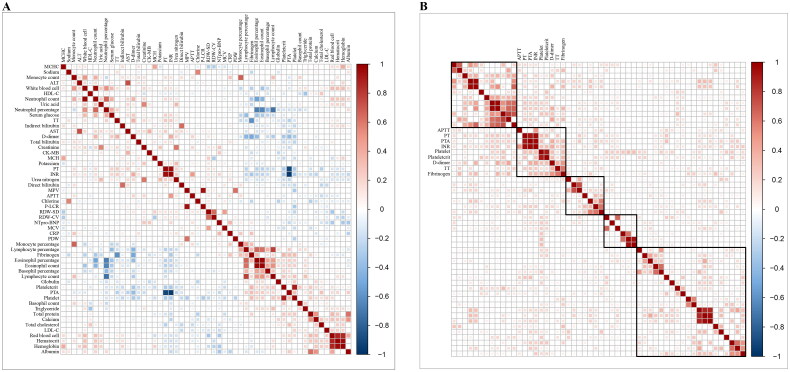
(A) Heatmap of spearman correlations. Red signifies positive, blue indicates negative correlations. Colour intensity denotes correlation strength, with non-significant results (*p* ≥ 0.05) left blank. (B) Matrix reordered using HCA for variables with *p* < 0.05 after absolute value treatment. Showing patterns within five classified clusters. HCA: hierarchical cluster analysis.

The results of Spearman correlation analysis are visually displayed in a heatmap diagram (Figure 1(A)). Hierarchical clustering as visualized by heat map analysis revealed groups of biological variables with analogous patterns. We systematically explored clustering schemes ranging from 2 to 12 clusters. Notably, within the 5 to 11 cluster classification, we consistently observed a prominent co-clustering of APTT, PT, PTA, INR, platelet, plateletcrit, D-dimer, TT and fibrinogen (Figure (1b), Supplementary Figure S2 (A-F)).

### Cox proportional hazards regression and interaction analysis

**Figure 2.**
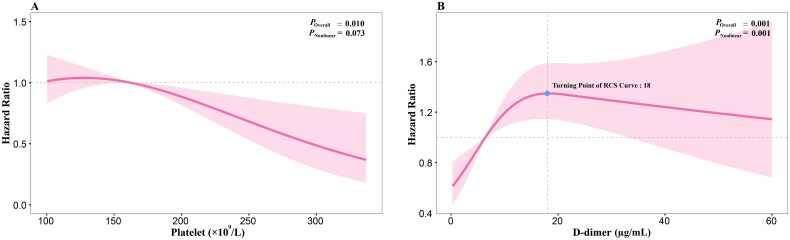
Restricted cubic spline. (A) Depicts platelet level’s restricted cubic spline for AAD in-hospital mortality. Hazard ratios adjusted for age, sex, smoking history, hypertension history, diabetes history, anatomical classification, onset time and hospital centres. (B) Displays D-dimer level’s restricted cubic spline for AAD in-hospital mortality, adjusting hazard ratios for age, sex, smoking history, hypertension history, diabetes history, anatomical classification, onset time and hospital centres. AAD: acute aortic dissection.

The Cox proportional hazards regression model revealed that platelet count, PT, PTA, TT, fibrinogen and D-dimer were associated with in-hospital mortality risk of AAD (*p* < 0.05). Platelet counts, PTA and fibrinogen exhibited linear negative correlations, suggesting lower levels associated with higher mortality risk (Figure 2a, Supplementary Figure S3 (A, B)). Conversely, PT and TT showed linear positive associations, indicating longer clotting times linked to increased mortality (Supplementary Figure S3 (C, D)). D-dimer displayed a nonlinear positive correlation, suggesting a threshold effect on mortality risk (Figure 2(B)). However, plateletcrit was found no significant association with mortality (*p* = 0.157) (see Supplementary Table S2). Patients were classified according to tertile cut-off points of platelet, PT, PTA, TT, fibrinogen and D-dimer levels. Separate tests were conducted to investigate how platelet levels interacted with other coagulative and fibrinolytic variables. Notably, a highly significant interaction was observed between platelet count and D-dimer level (*p* = 0.029) (see Supplementary Table S3).

### Independent association of platelet and D-dimer levels with in-hospital mortality in AAD

Mortality risk decreased monotonically across platelet-level categories. In the fully adjusted model, compared to the lowest platelet count category, the HR for the highest platelet level was 0.81 (95% CI: 0.65–1.02). Additionally, each standard deviation increment in platelet levels independently associates with a reduced hazard of in-hospital mortality (HR = 0.87, 95% CI: 0.78–0.97) (see Table 2). We found that a higher D-dimer level was associated with an increased risk of mortality in AAD, with a non-linear plateauing after reaching 18 µg/ml (*p* for non-linearity = 0.001). Specifically, relative to Q1, the hazard ratios (HRs) for Q2 and Q3 of D-dimer were 1.83 (95% CI: 1.29–2.62) and 2.16 (95% CI: 1.50–3.11), respectively (see Table 3). Below 18 µg/ml, the HR for each standard deviation increment was 1.24 (95% CI: 1.08, 1.43). From 18 µg/ml, the hazard ratio stagnates, and the standard deviations increase for the different batches. Restricted cubic spline of platelet and D-dimer levels for in-hospital mortality is shown (Figure 2).

**Table 2.** Parameters of platelet linearly associated with in-hospital mortality in AAD.

VAR	Tertile classification (HR, 95% CI)			Per SD increment in VAR
	Q1	Q2	Q3	
Platelet (×10^9^/L)	≤140 × 10^9^/L	140∼186 × 10^9^/L	≥186 × 10^9^/L	(HR, 95% CI)
Death/Total	235/847	190/838	144/840	
Model 1	1.00 (ref.)	0.78 (0.64, 0.94)	0.58 (0.47, 0.71)	0.76 (0.69, 0.83)
Model 2	1.00 (ref.)	0.81 (0.67, 0.99)	0.60 (0.49, 0.75)	0.77 (0.70, 0.85)
Model 3	1.00 (ref.)	0.91 (0.74, 1.12)	0.81 (0.65, 1.02)	0.87 (0.78, 0.97)

Model 1: No covariates were adjusted.

Model 2: Age, sex were adjusted.

Model 3: Age, sex, smoking history, hypertension history, diabetes history, anatomical classification, aorta diameter, onset time and hospital centres were adjusted.

AAD: acute aortic dissection; HR: hazard ratio; CI: confidence interval; SD: standard deviation; VAR: variable.

**Table 3.** Parameters of D-dimer non-linearly associated with In-hospital mortality in AAD.

VAR	Tertile classification (HR, 95% CI)			Per SD increment in VAR (HR, 95% CI)	
	Q1	Q2	Q3		
D-dimer (µg/mL)	≤3.4 µg/mL	3.4∼14.6 µg/mL	≥14.6 µg/mL	<18.0 µg/mL	≥18 µg/mL
Death/total	56/563	129/566	182/565	208/1198	159/496
Model 1	1.00 (ref.)	2.33 (1.70, 3.19)	3.39 (2.51, 4.57)	1.46 (1.30, 1.64)	0.91 (0.73, 1.14)
Model 2	1.00 (ref.)	2.33 (1.70, 3.19)	3.36 (2.89, 4.54)	1.46 (1.30, 1.64)	0.91 (0.72, 1.14)
Model 3	1.00 (ref.)	1.83 (1.29, 2.62)	2.16 (1.50, 3.11)	1.24 (1.08, 1.43)	1.13 (0.82, 1.56)

Model 1: No covariates were adjusted.

Model 2: Age, sex were adjusted.

Model 3: Age, sex, smoking history, hypertension history, diabetes history, anatomical classification, aorta diameter, onset time and hospital centres were adjusted.

AAD: acute aortic dissection; HR: hazard ratio; CI: confidence interval; SD: standard deviation; VAR, variable.

### Association of the combination of platelet and D-dimer levels with in-hospital mortality in AAD

**Figure 3.**
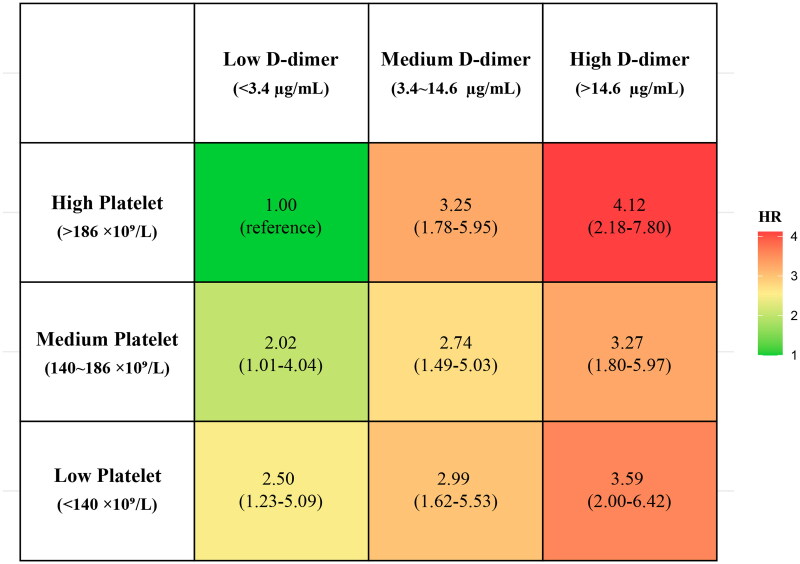
Association of the combination of platelet count and D-dimer levels with in-hospital mortality in AAD. Employed multivariable cox regression with nine categories based on platelet and D-dimer levels. Adjusted hazard ratios for age, sex, smoking history, hypertension history, diabetes history, anatomical classification, onset time and hospital centres. AAD: acute aortic dissection.

We detected significant interactions between platelet category and D-dimer category for in-hospital mortality in AAD (*p* = 0.029). Furthermore, compared with high platelet (>186 × 10^9^/L) and low D-dimer level (<14.6 µg/mL), HR of both high platelet (>186 × 10^9^/L) and high D-dimer level (>14.6 µg/mL) for mortality was 4.12 (95% CI: 2.18–7.80), whereas those of thrombocytopenia (<140 × 10^9^/L) and high D-dimer level (>14.6 µg/mL) for mortality was 3.59 (95% CI: 2.00–6.42) (Figure 3).

### Sensitivity analysis

Several sensitivity analyses were conducted to examine whether various stratified Cox proportional hazards analyses showed consistent results across various demographic and clinical factors, including age, sex, hypertension history, smoking history and onset time. Following comprehensive adjustments for age, sex, smoking history, hypertension history, diabetes history, anatomical classification, aorta diameter, onset time and hospital centres, the associations between platelet levels and in-hospital mortality in AAD patients were explored and are presented in Supplementary Table S4. Similarly, the relationship between D-dimer levels and in-hospital mortality was assessed within different subgroups, and the results are summarized in Supplementary Table S5. These analyses demonstrated the robustness of the observed associations within specific strata.

## Discussion

In this multicentre retrospective study, after analyzing platelet and all coagulation-fibrinolysis system variables, we observed that changes in platelet count, PT, PTA, INR, TT and D-dimer levels were correlated with the risk of in-hospital mortality in AAD following adjustments for potential confounders. Furthermore, amongst these variables, a significant mutual interaction was observed only between platelet count and D-dimer level (*p* = 0.029). The combined effect of thrombocytopenia and elevated D-dimer levels significantly increases the risk of in-hospital mortality in patients with AAD. This interaction shows a stronger predictive effect on clinical outcomes compared to individual variables or two noninteracting variables. This is the first comprehensive study of platelet and coagulation-fibrinolysis system variables and risk of in-hospital mortality in large-sample AAD patients.

AAD remains a life-threatening condition. To identify high-risk patients prone to adverse outcomes, researchers have explored various predictors. Factors such as advanced age, female gender, hypotension, renal insufficiency and incomplete false lumen thrombosis are associated with an increased risk of adverse events in AAD cases [23–26]. However, some factors mentioned above frequently lack accessibility at the bedside, are time-consuming to measure, or are challenging to assess accurately. For example, while aortic CT angiography offers a direct and reliable assessment of AAD, the procedure poses a high risk during patient transportation, can potentially impair renal function due to contrast agent use, and is expensive, making repeated examinations impractical. Therefore, it is necessary to identify convenient, accessible and reliable biomarkers to predict AAD outcomes during hospitalization. Our study has developed a risk assessment tool based on simple blood parameters, meeting the clinical needs outlined above.

Alterations in platelet and coagulation-fibrinolysis system variables accompany the entire course of AAD occurrence, development and outcome. Some of these variables are applicable not only for diagnosing AAD but also for assessing its prognosis. Platelets play a crucial role in the process of AAD. When AAD begins, collagen exposure triggers the release of tissue factors, leading to the formation of the false lumen, prompting immediate activation and aggregation of platelets [27,28]. The resultant platelet consumption might lead to declining platelet counts. In addition, highly activated platelets release inflammatory mediators into the local microenvironment, exacerbating the systemic inflammatory status [29]. Therefore, a decreased platelet count might indicate a consumption process following inflammation and thrombosis in AAD [30,31]. Previous studies have indicated that lower platelet counts on admission are associated with an increased risk of mortality in AAD patients with or without procedural intervention [14,23,24]. The current study further confirms the value of thrombocytopenia in predicting adverse outcomes in AAD patients of different anatomical classifications. In addition, we also observed a linear correlation between PT, PTA, TT and fibrinogen and in-hospital mortality in AAD patients. For the first time, correlation analysis and hierarchical cluster analysis were employed to choose research variables in AAD patients. This method not only mitigated the impact of human factors but also guaranteed cohesive combinations of biological variables.

Our findings also identified a non-linear correlation between D-dimer levels and the risk of in-hospital mortality in AAD, characterized by a threshold effect at 18 µg/ml. In simpler terms, as D-dimer levels increased, the risk of in-hospital mortality in patients gradually increased. However, once they exceeded 18 µg/ml, we observed a levelling off in the upward trend. D-dimer, a product of degraded cross-linked fibrin clots, serves as a biomarker in the fibrinolysis process. During the progression of AAD, in addition to platelet aggregation and coagulation activation, the fibrinolysis system is also triggered. High D-dimer levels reflect the hypercoagulability state and indicate high rates of platelet consumption, to a certain extent reflecting the severity of the condition [32,33]. D-dimer levels might elevate within just 1 h following the commencement of acute AD, and the rapid D-dimer assay can be concluded within 10 min. Therefore, D-dimer holds significant value in both differential diagnosis and predicting prognosis. More importantly, a significant mutual interaction was observed between platelet count and D-dimer level. In the subgroup analysis, compared to the reference group, patients with thrombocytopenia and high D-dimer levels exhibited an HR of 3.59 (95% CI: 2.00–6.42).

The observed link between the combined factors of platelet count and D-dimer levels with in-hospital mortality in AAD patients can be attributed to several interrelated mechanisms: (1) Reflective extent of vascular injury: both low platelet count and high D-dimer levels may indicate a more extensive vascular injury. A larger or more complicated aortic dissection could lead to greater activation of the coagulation cascade and more extensive platelet consumption, resulting in thrombocytopenia and elevated D-dimer levels; (2) Marker of hypercoagulability and fibrinolysis: This combination suggests a state of hypercoagulability and active fibrinolysis. This dynamic state can be particularly perilous, potentially leading to complications such as disseminated intravascular coagulation (DIC), organ ischaemia, or significant bleeding; (3) Indicative of secondary complications: AAD patients with this biomarker profile may be more susceptible to secondary complications, including enhanced systemic inflammatory response and organ dysfunction, both of which significantly increase the risk of mortality. Therefore, this interaction of two variables serves as a more comprehensive indicator of the severity of AAD and the body’s response to vascular injury.

The study has several potential limitations. First, this survey is a retrospective analytical study, inevitably, it confronts potential biases resulting from incomplete data sets. Second, given that the present study was performed in the Chinese Han population, there is a necessity for further exploration to validate these outcomes across various racial/ethnic groups for broader applicability. Third, we thoroughly considered the potential influences of centre and cluster effects on our results in our statistical analyses. By controlling for hospital centres as a confounding factor, we aimed to mitigate their impact. However, residual influences may persist, potentially impacting the generalizability of our findings. Fourth, as is the case with all observational studies, it was not possible to determine the extent to which these associations might be causal. Fifth, due to the retrospective nature of the study, it was not possible to definitively ascertain the specific causes of death in patients, including whether organ failure was a primary factor in mortality. This limitation is intrinsic to the nature of retrospective research, and prospective cohort studies in the future will be better positioned to address these questions. Finally, due to the lack of long-term follow-up data, we cannot assess the impact of changes in coagulation and fibrinolytic system variables on the long-term prognosis of AAD. Future prospective studies are necessary to provide more robust clinical evidence. Notwithstanding these limitations, it is our strong contention that this study presents a simple, expeditious and efficacious stratification tool for clinicians, specifically emergency physicians, enabling a more precise assessment of mortality risk in individuals with acute aortic dissection. This facilitates expedited patient care and reduces the likelihood of physician-patient conflicts. In regions with large geographical areas and uneven distributions of medical resources, the significance of such an assessment tool is particularly critical. In addition, this study also highlights the importance of monitoring platelet and coagulation-fibrinolysis system variables in AAD patients. While there have been reports of interventions in AAD patients with DIC, such as the use of heparin anticoagulation and supplementation of coagulation factors, there is still no research reporting on whether proactive interventions in platelet and coagulation fibrinolysis systems can improve patient prognosis [34,35]. This suggests a potential new direction for AAD treatment, which could be explored in future animal experiments.

## Conclusion

Our study emphasizes the significance of platelet and coagulation-fibrinolysis system variables in identifying AAD patients at high risk of poor outcomes. Moreover, the combined effect of thrombocytopenia and elevated D-dimer levels significantly increases the risk of in-hospital mortality in AAD. We recommend the prompt and repeated assessment of platelet counts and coagulation-fibrinolysis system variables upon admission, employing them as pivotal biomarkers for risk stratification in AAD patients.

## Supplementary Material

Supplementary Figure S1.tif

Clean copy - Supplementary_Tables.docx

Supplementary Figure S2.tif

Supplementary Figure S3.tif

## Data Availability

The datasets used and analysed during the current study are available from the corresponding author on reasonable request.
