# Supplementary figures and images for: Association of thrombocytopenia and D-dimer elevation with in-hospital mortality in acute aortic dissection
Source: Ann Med. 2025 Mar 21;57(1):2478477. doi: 10.1080/07853890.2025.2478477 (PMC11934191; doi:10.1080/07853890.2025.2478477)

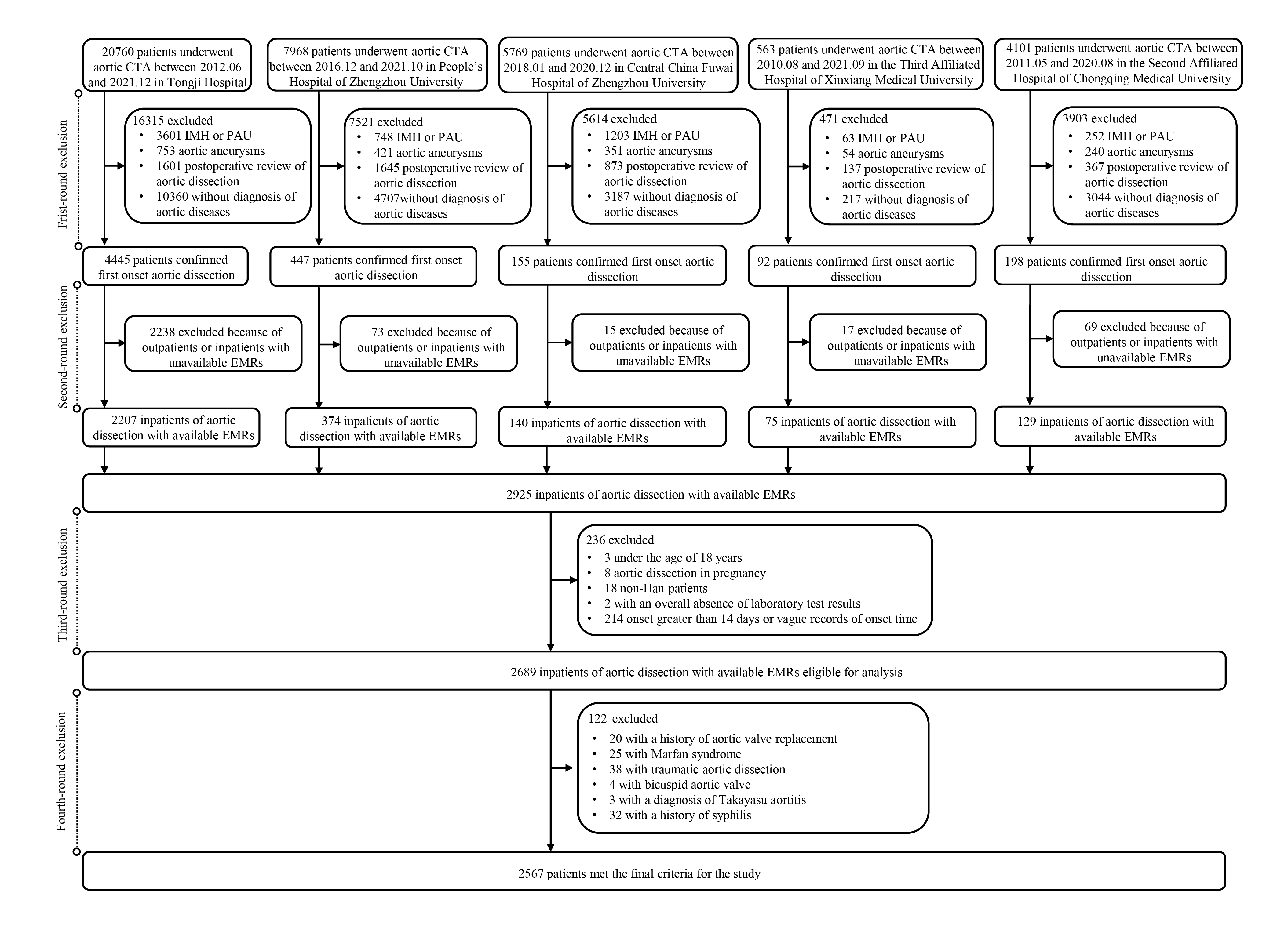

Supplement: Supplementary Figure S1.tif [file IANN_A_2478477_SM9388.tif]

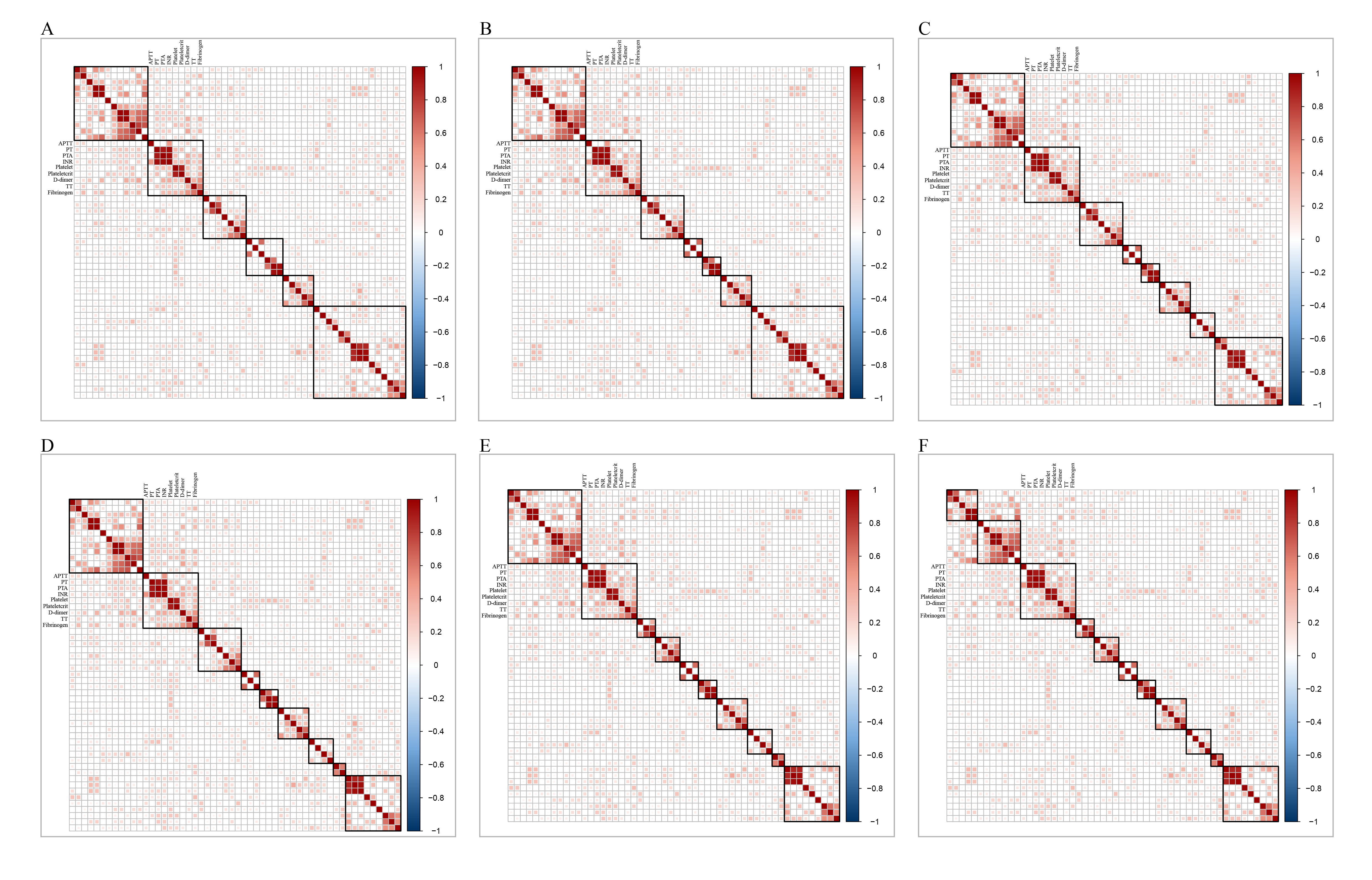

Supplement: Supplementary Figure S2.tif [file IANN_A_2478477_SM9386.tif]

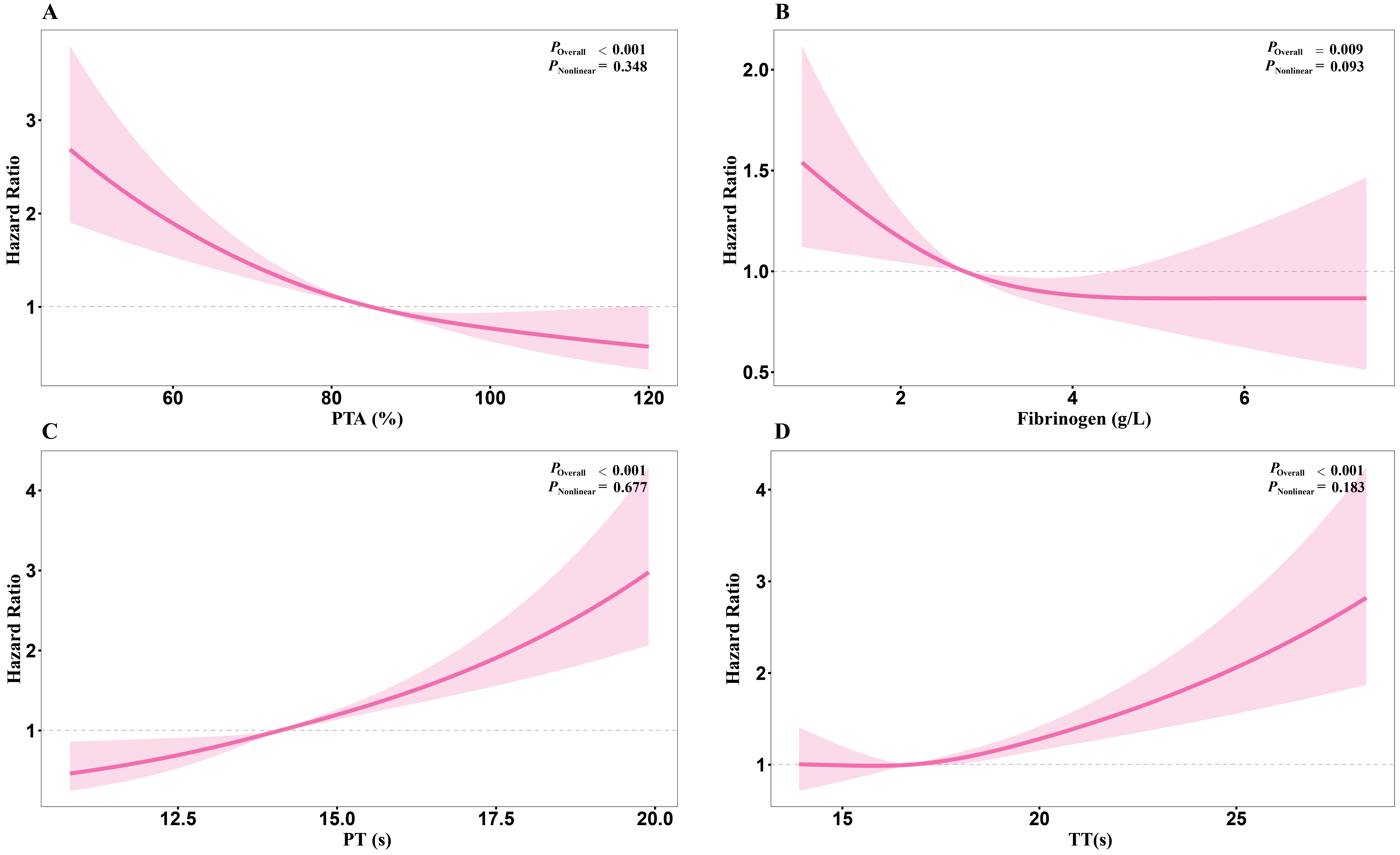

Supplement: Supplementary Figure S3.tif [file IANN_A_2478477_SM9385.tif]
