# Supplementary material for: Association of thrombocytopenia and D-dimer elevation with in-hospital mortality in acute aortic dissection
Source: Ann Med. 2025 Mar 21;57(1):2478477. doi: 10.1080/07853890.2025.2478477 (PMC11934191; doi:10.1080/07853890.2025.2478477)
Supplement: Clean copy - Supplementary_Tables.docx [file IANN_A_2478477_SM9387.docx]

**Supplementary Tables**

| **Table S1. Baseline Characteristics of Patients with AAD Stratified by D-dimer Levels** | | | |  |
| --- | --- | --- | --- | --- |
| **Characteristics** | ≤3.4 µg/mL | 3.4～14.6 µg/mL | ≥14.6 µg/mL |  |
| Age, median (IQR), years | 54.0 (45.0, 63.0) | 54.0 (45.0, 63.0) | 56.0 (50.0, 63.0) |  |
| Sex, Male, n (%) | 455 (80.1%) | 452 (80.6%) | 443 (78.4%) |  |
| Anatomical classification |  |  |  |  |
| DeBakey I, n (%) | 142 (25.0%) | 263 (46.9%) | 396 (70.1%) |  |
| DeBakey II, n (%) | 44 (7.7%) | 33 (5.9%) | 15 (2.7%) |  |
| DeBakey IIIa, n (%) | 34 (6.0%) | 8 (1.4%) | 6 (1.1%) |  |
| DeBakey IIIb, n (%) | 307 (54.0%) | 243 (43.3%) | 142 (25.1%) |  |
| Isolated abdominal AAD, n (%) | 41 (7.2%) | 14 (2.5%) | 6 (1.1%) |  |
| History |  |  |  |  |
| Smoking, n (%) | 273 (48.1%) | 245 (43.7%) | 230 (40.7%) |  |
| Hypertension, n (%) | 398 (70.1%) | 417 (74.3%) | 391 (69.2%) |  |
| Diabetes, n (%) | 32 (5.6%) | 8 (1.4%) | 18 (3.2%) |  |
| Larger aorta diameter(≥5.5 cm), n (%) | 12 (2.1%) | 15 (2.7%) | 6 (1.1%) |  |
| Onset time |  |  |  |  |
| < 24 hours, n (%) | 249 (43.8%) | 340 (60.6%) | 436 (77.2%) |  |
| 1-7 days, n (%) | 267 (47.0%) | 210 (37.4%) | 126 (22.3%) |  |
| 8-14 days, n (%) | 52 (9.2%) | 11 (2.0%) | 3 (0.5%) |  |
| Hospital centers, n (%) |  |  |  |  |
| Tongji Hospital | 340 (59.9%) | 456 (81.3%) | 501 (88.7%) |  |
| Henan Provincial People's Hospital | 105 (18.5%) | 61 (10.9%) | 33 (5.8%) |  |
| Fuwai Central China Cardiovascular Hospital | 27 (4.8%) | 26 (4.6%) | 14 (2.5%) |  |
| Third Affiliated Hospital of Xinxiang Medical University | 31 (5.5%) | 15 (2.7%) | 2 (0.4%) |  |
| Second Affiliated Hospital of Chongqing Medical University | 65 (11.4%) | 3 (0.5%) | 15 (2.7%) |  |
| Hospital mortality, n (%) | 56 (9.9%) | 129 (23.0%) | 182 (32.2%) |  |
| Hospitalization time, median (IQR), days | 12.0 (6.0, 19.0) | 11.0 (4.0, 21.0) | 11.0 (2.0, 22.0) |  |
| Platelet, median (IQR), ×10^9^/L | 186.0 (148.0, 230.0) | 164.0 (133.0, 200.0) | 145.0 (118.0, 176.0) |  |
| Plateletcrit, median (IQR), % | 0.2 (0.2, 0.2) | 0.2 (0.1, 0.2) | 0.2 (0.1, 0.2) |  |
| APTT, median (IQR), s | 35.8 (32.2, 39.8) | 36.5 (33.3, 40.4) | 39.0 (35.5, 43.0) |  |
| TT, median (IQR), s | 16.3 (15.3, 17.2) | 16.2 (15.4, 17.1) | 18.1 (16.9, 19.9) |  |
| PT, median (IQR), s | 13.5 (12.6, 14.2) | 14.0 (13.2, 14.7) | 14.6 (13.9, 15.7) |  |
| PTA, median (IQR), % | 91.0 (83.3, 103.0) | 87.0 (78.0, 97.0) | 79.0 (69.0, 88.0) |  |
| INR, median (IQR) | 1.1 (1.0, 1.1) | 1.1 (1.0, 1.2) | 1.2 (1.1, 1.3) |  |
| Fibrinogen, median (IQR), g/L | 3.4 (2.7, 4.8) | 2.9 (2.3, 3.7) | 2.1 (1.6, 2.6) |  |
| Continuous variables are represented as median (IQRs) and categorical variables as number (%). | | | |  |
| Abbreviations: IQR, interquartile range; AAD, acute aortic dissection; APTT, Activated Partial Thromboplastin Time; TT, Thrombin Time; PT, Prothrombin Time; PTA, Prothrombin Activity; INR, International Normalized Ratio. | | | |  |
|  |  |  |  |  |

| **Table S2. P-value of RCS-fitted Cox Models for Various Biological Variables** | | |
| --- | --- | --- |
| **Characteristics** | **P-Overall** | **P-Nonlinear** |
| PT | <0.001 | 0.677 |
| PTA | <0.001 | 0.348 |
| Platelet | 0.010 | 0.073 |
| Plateletcrit | 0.157 | 0.369 |
| D-dimer | 0.001 | 0.001 |
| TT | <0.001 | 0.183 |
| Fibrinogen | 0.009 | 0.093 |
| Abbreviations: RCS, Restricted Cubic Spline; PT, Prothrombin Time; PTA, Prothrombin Activity; TT, Thrombin Time. | | |

| **Table S3. P-values of Interaction of Platelet with Other Coagulation and Fibrinolysis Parameters** | | |
| --- | --- | --- |
| **Characteristics** | | **P-interaction** |
| Platelet | PT | 0.097 |
|  | PTA | 0.611 |
|  | D-dimer | 0.029 |
|  | TT | 0.503 |
|  | Fibrinogen | 0.705 |
| Abbreviations: PT, Prothrombin Time; PTA, Prothrombin Activity; TT, Thrombin Time. | | |

| **Table S4. Stratified analyses of the associations of platelet level with In-hospital mortality of AAD** | | | |
| --- | --- | --- | --- |
| **Variables** | **Deaths/N** | **Per SD increment** | ***P* value for interaction** |
| Age, years |  |  |  |
| < 60 | 371/1703 | 0.86 (0.76, 0.98) | 0.528 |
| ≥ 60 | 218/864 | 0.85 (0.71, 1.00) |  |
| Sex |  |  |  |
| Female | 135/531 | 0.85 (0.70, 1.03) | 0.352 |
| Male | 454/2036 | 0.88 (0.78, 0.99) |  |
| Smoking history |  |  |  |
| No | 388/1530 | 0.93 (0.83, 1.05) | 0.187 |
| Yes | 201/1037 | 0.73 (0.60, 0.90) |  |
| Hypertension history |  |  |  |
| No | 228/819 | 0.99 (0.84, 1.16) | 0.374 |
| Yes | 361/1748 | 0.81 (0.71, 0.93) |  |
| Onset time |  |  |  |
| Hyperacute (< 24 hours) | 392/1530 | 0.94 (0.84, 1.05) | 0.505 |
| Acute (1-14 days) | 197/1037 | 0.79 (0.65, 0.96) |  |
| Data was represented as HR (95% CI) with adjusted for age, sex, smoking history, hypertension history, diabetes history, anatomical classification, aorta diameter, onset time and hospital centers. | | | |
| Abbreviation: AAD, acute aortic dissection; SD, standard deviation; HR, Hazard Ratio; CI, Confidence Interval. | | | |

| **Table S5. Stratified analyses of the associations of d-dimer level with In-hospital mortality of AAD** | | | |
| --- | --- | --- | --- |
| **Variables** | **Deaths/N** | **Per SD increment** | ***P* value for interaction** |
| Age, years |  |  |  |
| < 60 | 371/1703 | 1.12 (1.01, 1.24) | 0.423 |
| ≥60 | 218/864 | 0.81 (0.32, 1.06) |  |
| Sex |  |  |  |
| Female | 135/531 | 0.24 (0.00, 52.06) | 0.523 |
| Male | 454/2036 | 1.08 (0.96, 1.21) |  |
| Smoking history |  |  |  |
| No | 388/1530 | 0.75 (0.33, 1.74) | 0.444 |
| Yes | 201/1037 | 1.14 (0.99, 1.31) |  |
| Hypertension history |  |  |  |
| No | 228/819 | 0.71 (0.15, 3.43) | 0.561 |
| Yes | 361/1748 | 1.06 (0.96, 1.17) |  |
| Onset time |  |  |  |
| Hyperacute (< 24 hours) | 392/1530 | 1.17 (1.01, 1.35) | 0.547 |
| Acute (1-14 days) | 197/1037 | 0.89 (0.49, 1.61) |  |
| Data was represented as HR (95% CI) with adjusted for age, sex, smoking history, hypertension history, diabetes history, anatomical classification, aorta diameter, onset time and hospital centers. | | | |
| Abbreviations: AAD, acute aortic dissection; SD, standard deviation; HR, Hazard Ratio; CI, Confidence Interval. | | | |
